# Analysis of heterogeneous growth changes in longitudinal height of children

**DOI:** 10.1186/s41043-023-00425-y

**Published:** 2023-08-08

**Authors:** Senahara Korsa Wake, Temesgen Zewotir, Essey Kebede Muluneh

**Affiliations:** 1 College of Science, Bahir Dar University, Bahir Dar, Ethiopia; 2 College of Natural and Computational Sciences, Ambo University, Ambo, Ethiopia; 3 School of Mathematics, Statistics and Computer Science, University of KwaZulu-Natal, Durban, South Africa; 4 School of Public Health, Bahir Dar University, Bahir Dar, Ethiopia

**Keywords:** Latent class growth model, Latent basis model, Latent growth analysis, Multiple-group growth model, Observed heterogeneity, Unobserved heterogeneity

## Abstract

**Background:**

There have been methodologies developed for a wide range of longitudinal data types; nevertheless, the conventional growth study is restricted if individuals in the sample have heterogeneous growth trajectories across time. Using growth mixture modeling approaches, we aimed to investigate group-level heterogeneities in the growth trajectories of children aged 1 to 15 years.

**Method:**

This longitudinal study examined group-level growth heterogeneities in a sample of 3401 males and 3200 females. Data were analyzed using growth mixture modeling approaches.

**Results:**

We examined different trajectories of growth change in children across four low- and middle-income countries using a data-driven growth mixture modeling technique. The study identified two-group trajectories: the most male samples group (*n* = 4260, 69.7%) and the most female samples group (*n* = 2341, 81.6%). The findings show that the two groups had different growth trajectories. Gender and country differences were shown to be related to growth factors; however, the association varied depending on the trajectory group. In both latent groups, females tended to have lower growth factors (initial height and rate of growth) than their male counterparts. Compared with children from Ethiopia, children from Peru and Vietnam tended to exhibit faster growth in height over time: In contrast, children from India showed a lower rate of change in both latent groups than that of children from Ethiopia.

**Conclusions:**

The height of children in four low- and middle-income countries showed heterogeneous changes over time with two different groups of growth trajectories.

## Introduction

Describing how individuals vary over time is one of the main focuses in the study of development [1]. Repeated measurements are commonly used to study individual and population growth processes of stability and change over time [2]. Thus, selecting analytical methodologies that capture growth changes is an important point in longitudinal data analysis. Growth curve modeling has offered a set of techniques that are useful for modeling between-individual variations and within-individual change in growth [3]. In analyzing the change process, it is reasonable to assume that not everyone changes in similar trajectories. There may be underlying classes for each individual, with each class having its own set of parameters guiding the change process, or possibly following distinct functional forms of change entirely [4]. In such instances, the application of typical longitudinal data analysis techniques may not give appropriate results.

Growth modeling techniques, such as mixed-effects and latent growth models, assume that the sample is drawn from a single population with a single set of parameters (e.g., means, variances, covariance) [5, 6]. For a single population, covariates that explain part of the variances of the growth parameters (e.g., intercept and slope) can reflect observed variation in growth curve models. However, in the situation of unobserved heterogeneity, the assumption of a single population underlying the growth curves must be relaxed [2]. In contrast, multiple-group latent growth models can enable simultaneous modeling of change for multiple observed groups, in which parameters describing growth patterns are examined to see if they are invariant across groups. However, they require prior information about the group membership of an individual and they assume various growth trajectories can only be examined within subgroups that have observed identity variables (e.g., gender, etc.). Furthermore, when the group membership of an individual is unknown, unobserved subgroups of individuals may exist within the population, which may exhibit variability in their latent trajectories. Growth mixture modeling is a viable approach for identifying multiple unique trajectories in different unobserved subgroups. It is appropriate when subgroups of individuals with different trajectories are expected and individual group membership is unknown a priori [5–7].

Growth mixture models can be considered as an extension of the multiple-group latent growth model. An individual's group membership can be generated from the information of the estimated probabilities of latent class [2]. Hence, the growth mixture model refers to a model with categorical latent variables that reflect subgroups where group membership is unknown, and it is known as a finite mixture model. The mixture represents various latent trajectory classes [8]. Therefore, this study aims to present the growth mixture modeling approach to identify groups of children with different growth trajectories of physical height and to examine growth differences across different groups of individuals.

## Methods

### Study sample

Longitudinal height data were obtained from the Young Lives cohort study carried out in Ethiopia, India, Peru and Vietnam from 2002 to 2016. The Young Lives study is a 15-year longitudinal study of the changing nature of childhood poverty designed to collect information on children growing up in four low- and middle-income countries. The Young Lives study employed multistage sampling techniques with the first stage involving a selection of sentinel locations from each country. Sentinel site monitoring is a public health concept that entails a purposive sampling of a small number of settings that are thought to reflect a specific population or area, and then being studied uniformly at relatively wide ranges. Following that, 20 sentinel sites were selected at non-random in each study country. Following the selection of 20 sentinel sites, households with children in the appropriate age groups were chosen at random. This technique, which was implemented in 2002, resulted in the selection of 2,000 infants (ages 6 to 18 months) at random and considered as a younger cohort. Simultaneously, 1,000 older children (aged 7 to 8 years) were chosen at random in the same sites and considered as older cohort [9, 10]. Details regarding sampling and participant recruitment have been discussed in previously published works [9, 11].

The qualitative and quantitative survey data were gathered in five rounds. The first survey round was carried out in 2002 when the children were on average one year of age (younger cohort) and eight years of age (older cohort), the second survey was carried out in 2006, the third was in 2009, the fourth was in 2013, and the fifth was carried out in 2016. The anthropometric data were collected as quantitative data. Details regarding sampling and participant recruitment were discussed in previously published studies [12–18]. From a younger cohort, a total of 3401 males and 3200 females with measured height 5 times from ages 1 to 15 years were included in this study. The Young Lives data are publicly available and can be accessed from http://www.younglives.org.uk/.

### Mixture models with Known group Membership

The goal of growth curve modeling is to characterize and test inter-individual variations in intra-individual change [19]. Latent growth models comprise the prediction of one latent class which is defined as a single population mean trajectory or multiple latent classes which are defined as one mean trajectory per class. Each latent group can be thought of as a collection of subjects that have similar growth trajectories that are not observed in the data [20]. In conventional growth modeling techniques, the sample is taken from a single population with a single set of growth parameters. A growth curve model can be extended to investigate variations in outcome growth among known latent multiple groups [5, 20–22]. Hence, multiple-group latent growth curve modeling required prior knowledge about an individual's group membership and the number of groups; for instance, gender can be divided into male group and female group, which are known and observed groups. Independent growth models are described for each group, which indicate the pattern of change, the mean change and the extent of between-group variations in the amount of change [5]. Multiple-group latent growth models [3] for *g* groups (*g* = 1, 2, …, *G*) can be expressed as follows:

1 $$y_{ti}^{{\left( {\text{g}} \right)}} = \alpha_{i}^{{\left( {\text{g}} \right)}} + \beta_{i}^{{\left( {\text{g}} \right)}} {\uplambda }_{t}^{{\left( {\text{g}} \right)}} + \varepsilon_{ti}$$

where

2 $$\alpha_{i}^{{\left( {\text{g}} \right)}} = \mu_{\alpha }^{{\left( {\text{g}} \right)}} + e_{\alpha }^{{\left( {\text{g}} \right)}}$$

3 $$\beta_{i}^{{\left( {\text{g}} \right)}} = \mu_{\beta }^{{\left( {\text{g}} \right)}} + e_{\beta }^{{\left( {\text{g}} \right)}}$$

Equation (1) can be expressed in the matrix form of structural equation modeling as:

4 $${\varvec{y}}^{{\left( {\text{g}} \right)}} = \eta^{{\left( {\text{g}} \right)}} {\Lambda }^{{\left( {\text{g}} \right)}} + \varepsilon^{{\left( {\text{g}} \right)}}$$

where

$${\varvec{y}}^{{\left( {\text{g}} \right)}} = \left[ {\begin{array}{*{20}c} {y_{1i}^{{\left( {\text{g}} \right)}} } \\ {y_{2i}^{{\left( {\text{g}} \right)}} } \\ {\begin{array}{*{20}c} {\begin{array}{*{20}c} {y_{3i}^{{\left( {\text{g}} \right)}} } \\ \vdots \\ \end{array} } \\ {y_{Ti}^{{\left( {\text{g}} \right)}} } \\ \end{array} } \\ \end{array} } \right],{\Lambda }^{{\left( {\text{g}} \right)}} = \left[ {\begin{array}{*{20}c} {\begin{array}{*{20}c} 1 \\ 1 \\ {\begin{array}{*{20}c} 1 \\ \vdots \\ \end{array} } \\ \end{array} } & {\begin{array}{*{20}c} 0 \\ 1 \\ {\begin{array}{*{20}c} {{\uplambda }_{3}^{{\left( {\text{g}} \right)}} } \\ \vdots \\ \end{array} } \\ \end{array} } \\ 1 & {{\uplambda }_{T}^{{\left( {\text{g}} \right)}} } \\ \end{array} } \right],\eta^{{\left( {\text{g}} \right)}} = \left[ {\begin{array}{*{20}c} {\alpha_{i}^{{\left( {\text{g}} \right)}} } \\ {\beta_{i}^{{\left( {\text{g}} \right)}} } \\ \end{array} } \right],\varepsilon^{{\left( {\text{g}} \right)}} = \left[ {\begin{array}{*{20}c} {\varepsilon_{1i}^{{\left( {\text{g}} \right)}} } \\ {\varepsilon_{2i}^{{\left( {\text{g}} \right)}} } \\ {\begin{array}{*{20}c} {\begin{array}{*{20}c} {\varepsilon_{3i}^{{\left( {\text{g}} \right)}} } \\ \vdots \\ \end{array} } \\ {\varepsilon_{Ti}^{{\left( {\text{g}} \right)}} } \\ \end{array} } \\ \end{array} } \right]$$

where $y_{ti}$ is the measured outcome for the *i*-th individual at time *t* = 1, 2, …, *T*, $\alpha_{i}^{{\left( {\text{g}} \right)}}$ and $\beta_{i}^{{\left( {\text{g}} \right)}}$ are latent variables representing the initial and growth rate for i-th individual in *g* group, respectively, $\Lambda_{t}$ represents the basis function for the slope component, and $\varepsilon_{ti}$ is a time-specific residual. Individual variations in growth over time are allowed in this model since the growth intercept $\alpha_{i}$ and slope $\beta_{i}$ vary across individuals, resulting in individually varying patterns for $y_{ti}$ over time.

### Mixture models with unknown group membership

In the multiple-group modeling approach, the group membership of each individual in the sample is known or observed. In some circumstances, observations may come from multiple populations with no way of identifying in which population a given observation belongs to. When group membership of individuals is not known, mixture modeling gives a way for estimating models. It is a popular data analysis technique for detecting heterogeneity in a group or population through multiple unobserved subgroups, characterizing change over time within each unobserved subgroup and investigating variations in change across unobserved subgroups [5, 23]. This technique simultaneously classifies individuals into different groups and estimates the latent curves in each group. Individuals' latent classes can be identified using longitudinal finite mixture models [8]. In finite mixture models, the individual under study is considered to be made up of various latent classes [24]. Each class represents a set of heterogeneous observations in which factors affect the response in different ways [20]. To capture heterogeneities in the population, the model separates individuals into different latent classes, each with its functional form for the trajectory. Individuals are then assigned to classes depending on the degree of their similarity [25] using posterior probabilities. The data are used to infer posterior probability for each individual in all classifications. To put it another way, for each individual, a posterior probability is calculated for each class, and the individual is categorized into the class with the highest posterior probability [22]. The model equation [3] can be formulated as:

5 $$y_{ti} = \mathop \sum \limits_{{{\text{g}} = 1}}^{G} P_{i}^{{\left( {\text{g}} \right)}} \left( {\alpha_{i}^{{\left( {\text{g}} \right)}} + \beta_{i}^{{\left( {\text{g}} \right)}} {\uplambda }_{t}^{{\left( {\text{g}} \right)}} + \varepsilon_{ti} } \right)$$

where $P_{i}^{{\left( {\text{g}} \right)}}$ is the probability that the i-th individual belongs to the *g*-th group such that $P_{i}^{{\left( {\text{g}} \right)}} \ge 0$, $\mathop \sum \limits_{{{\text{g}} = 1}}^{G} P_{i}^{{\left( {\text{g}} \right)}} = 1$, and *g* > 1. For continuous longitudinal outcomes, $y_{ti}$ is normally distributed with mean $\mu^{{\text{g}}}$ and variance–covariance ${\Sigma }^{{\text{g}}}$, $y_{ti} \sim MVN\left( {\mu^{{\text{g}}} ,{\Sigma }^{{\text{g}}} } \right)$. There are two sources of variation in ${\Sigma }^{{\text{g}}}$: between-individual variation given by random growth parameters (the intercept and slope) ($D^{{\text{g}}}$) and within-individual variation given by errors ($R^{{\text{g}}}$) [26].

The conventional growth modeling approach is not well-suited for investigating heterogeneous change in a single population. A growth mixture model considers that the group is made up of diverse subgroups of trajectories (i.e., a “mixture” of distributions), allowing for heterogeneous trajectories to be accommodated [22]. The first procedure in fitting a growth mixture model to the data is to estimate a conventional latent growth curve model to see the assumptions of homogeneous variance with a common growth function. Fitting a latent growth curve model to the data, in this case, enables the study to analyze the overall fit of the data to a particular shape of trajectory before accounting for trajectory heterogeneity. The presence of heterogeneous trajectories, or subgroups with diverse trajectories, is suggested by poor model fit and statistically significant variances in growth parameters [22]. To compare the fit of nested models, the likelihood ratio test can be utilized. The Bayesian information criterion (BIC) is used to determine the number of groups for non-nested models [21]. The number of groups is gradually increased until a minimum BIC is achieved [27].

**Fig. 1:**
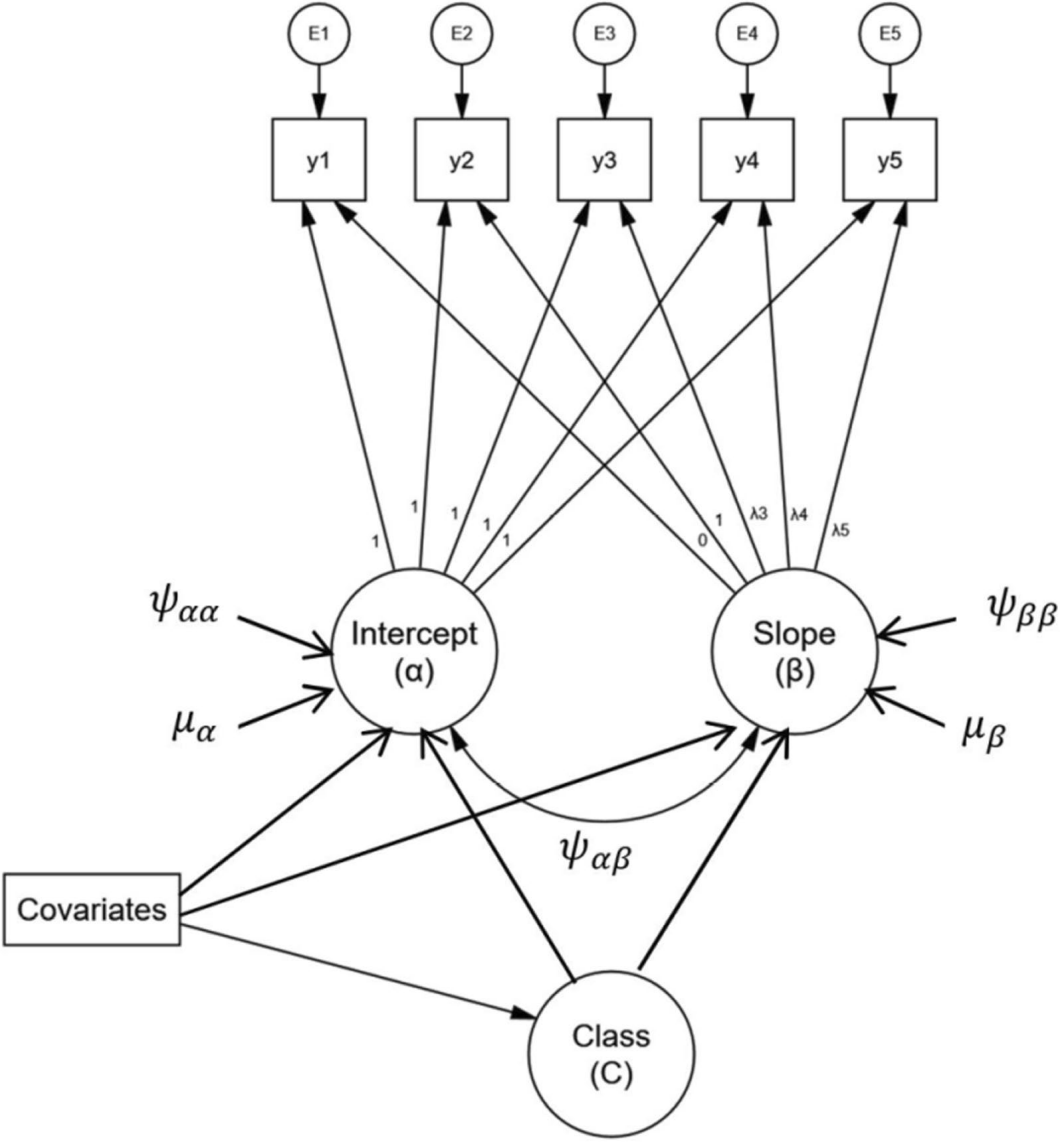
Conditional growth mixture model for five waves of data

As depicted in Fig. 1, a growth mixture model accounts for heterogeneity by including multiple groups or latent categorical variables (C) of individual trajectories in addition to components of the conventional growth model in the same modeling approach. Class C can be observed or unobserved/latent classes which represent the latent trajectory classes that underpin the latent growth variables.

## Results

This longitudinal study was designed to examine the group-specific trajectory in height growth from age 1 to 15 years using a mixture modeling technique. Four low- and middle-income countries were the group variable of interest of the study (Ethiopia: *n* = 1582, 24%, India: *n* = 1648, 25%, Peru: *n* = 1627, 24.6%, Vietnam: *n* = 1744, 26.4%). The summary of descriptive statistics for the endogenous variables and response frequencies for five measurement occasions is given in Table 1.

**Table 1:** Descriptive statistics of measurement of height for five measurement occasions by countries

		Height measurement occasion (age/year)				
Country/age (year)		Age 1	Age 5	Age 8	Age 12	Age 15
Ethiopia	Mean	71.375	104.467	121.348	141.785	156.851
	SD	5.336	5.087	5.744	6.572	7.117
	*n*	1582	1582	1582	1582	1582
India	Mean	72.159	104.648	119.488	141.197	155.937
	SD	4.802	4.550	5.485	6.757	7.351
	*n*	1648	1648	1648	1648	1648
Peru	Mean	71.781	104.899	120.782	143.654	157.590
	SD	4.524	5.820	5.556	7.040	7.076
	*n*	1627	1627	1627	1627	1627
Vietnam	Mean	72.415	105.319	121.539	144.849	159.136
	SD	4.194	4.886	5.777	7.762	7.098
	*n*	1744	1744	1744	1744	1744

Age 1,…, Age 15 are children’s height measurements at age 1,…age 15 years, respectively, *SD* Standard deviation

The height of the children was measured in five rounds from 2002 to 2016. The first survey round was carried out in 2002 when the children were on average one year of age, the second survey was carried out in 2006, the third in 2009, the fourth in 2013, and the fifth in 2016 when the children were on average 15 years of age. The younger children were tracked from one to fifteen years, at ages 1, 5, 8, 12 and 15 years. The mean height of children given in Table 1 further revealed that in all four countries, there are increasing tendencies of mean height from age one to age 15 years. However, there were variations in mean height changes over time among the four countries. On all measurement occasions from ages 1 to 15 years, children in Vietnam had the highest mean height, and children in Peru had the second highest mean height (Table 1).

### Latent basis growth mixture model with observed groups

The first step in modeling a growth mixture is to see how well a single-group basic latent growth model fits the data [6]. The basic latent growth curve model assumes the growth trajectories (Fig. 4) among children in Ethiopia, India, Peru and Vietnam differ only in their means intercepts, rate of growth or the impact of the covariates on these variables. However, the variation of intercept and slope was statistically significant ($\psi_{\alpha \alpha } = 12.94,p < 0.0001$, $\psi_{\beta \beta } = 0.07,p = 0.004$, respectively) and the functional form of the trajectory may also differ. The significant variation in the growth function between subjects indicates the presence of heterogeneity in longitudinal changes of height growth. To address these challenges, a single latent group was extended to observed multiple groups with considerably different growth patterns from the entire estimate. Hence, a separate functional form and data set were established for each group based on the residential country of the children.

**Fig. 2:**
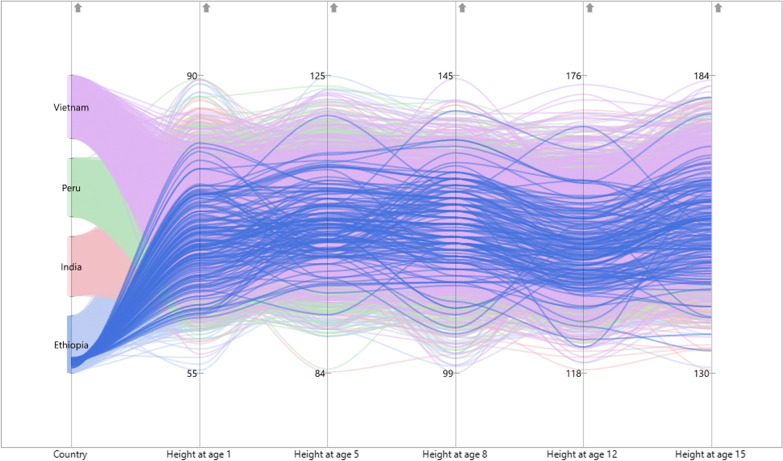
Growth trajectories for observed multiple groups

**Fig. 3:**
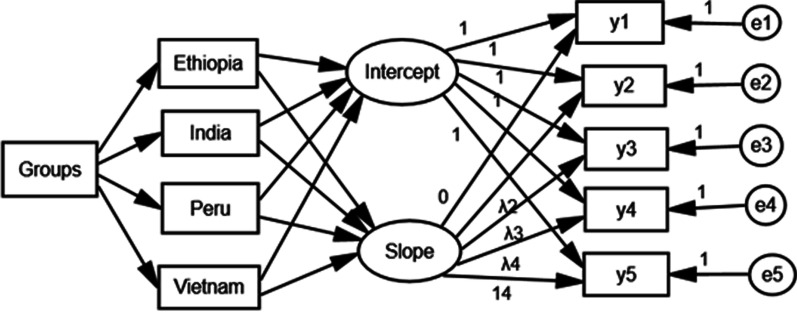
Multiple-group growth mixture models for height in Ethiopia, India, Peru and Vietnam

Figure 2 depicts the observed group patterns by countries, which represent different growth trajectories in different countries. In this case, we might identify four groups of growth trajectories (Ethiopia, India, Peru and Vietnam), each with its own predicted growth parameters. As shown in Fig. 2, the individual height of children in all four countries is separately displayed with nonlinear changes over time. The minimum and maximum heights in each measurement occasion were likewise displayed in Fig. 2. Figure 3 shows the structural equation framework for multiple-group unconditional growth mixture models for these observed groups.

Using multiple-group models, testing for multiple-group invariance across groups was performed. For instance, M1: unconstrained model (no equality constraints imposed across the groups), M2: testing group-varying in factor loadings, M3: testing group-varying in mean intercept, M4: testing group-varying in slope component, and M5: testing group-varying random variance ($\psi_{{\alpha_{g} }} ,\psi_{{\beta_{g} }}$) and covariance ($\psi_{{\alpha \beta_{g} }}$) in latent intercept and slope components. The likelihood ratio test was used to test the invariance nature of the growth components across the G groups due to the nested link between a model with constraints and an unconstrained model. Table 2 shows the chi-square difference test statistics for these models.

**Table 2:** A summary of fit statistics for multiple-group invariance tests

Model description	Comparative model	*X*^2^	df	${\varvec{\Delta}}$*X*^2^	${\varvec{\Delta}}$df	*p*-value
No equality constraints imposed (M1)	–	547.158	20	–	–	–
All factor loadings constrained equal (M2)	M2 vs M1	1005.86	29	458.699	9	0.000
Equal mean intercept (M3)	M3 vs M1	586.751	23	39.593	3	0.000
Equal rate of change (M4)	M4 vs M1	693.723	23	146.565	3	0.000
Equal random variance (M5)	M5 vs M1	569.571	23	22.413	3	0.000

$\Delta x^{2}$ = difference in chi-square values between models; $\Delta df$ = difference in the number of degrees of freedom between models; M1,…, M5 = Model 1, …, Model 5, respectively.

The chi-square difference ($\Delta x^{2}$) between the two nested models can be used to perform a significance test to see if the more restricted model fits the same as the less restrictive model without the restrictions. A significant $\Delta x^{2}$ indicates that the four classes differ in at least one of the growth parameters examined. Model 2 is a latent basis model in which the loading factors of $\lambda_{1} = 0,\lambda_{5} = 14$ constraints were applied to all groups. The rest loading factors, $\lambda_{2}$,$\lambda_{3}$ and $\lambda_{4}$, are unconstrained across all groups to permit different functional forms of trajectories for each group. A chi-square difference test of 458.699 with *p* = 0.000 in Table 2 indicates that at least one group has distinct growth patterns. Model 3 and 4 examined group-varying in mean intercept and rate of change, respectively. The small value of *p* = 0.000 suggests that at least one group has a different mean intercept and rate of change than other groups. The parameter estimates of these models are presented in Table 3.

**Table 3:** Parameter estimates of multiple-group analysis of longitudinal height data for Ethiopia, India, Peru and Vietnam

Group	Parameter	Estimate	SE	CR	*p*-value
Ethiopia	Intercept ($\alpha$)	71.376	0.136	524.896	***
	Rate of change ($\beta$)	6.105	0.013	465.808	***
	Random intercept variance ($\psi_{\alpha \alpha }$)	12.569	0.938	13.397	***
	Random slope variance ($\psi_{\beta \beta }$)	0.112	0.01	10.944	***
	Covariance ($\psi_{\alpha \beta }$)	0.061	0.08	0.762	0.446
India	Intercept ($\alpha$)	72.15	0.122	590.749	***
	Rate of change ($\beta$)	5.984	0.013	463.608	***
	Random intercept variance ($\psi_{\alpha \alpha }$)	12.663	0.748	16.936	***
	Random slope variance ($\psi_{\beta \beta }$)	0.087	0.009	9.349	***
	Covariance ($\psi_{\alpha \beta }$)	0.116	0.067	1.732	0.083
Peru	Intercept ($\alpha$)	71.768	0.12	597.644	***
	Rate of change ($\beta$)	6.129	0.012	528.918	***
	Random intercept variance ($\psi_{\alpha \alpha }$)	12.945	0.799	16.194	***
	Random slope variance ($\psi_{\beta \beta }$)	0.051	0.008	6.386	***
	Covariance ($\psi_{\alpha \beta }$)	0.463	0.063	7.418	***
Vietnam	Intercept ($\alpha$)	72.399	0.108	669.674	***
	Rate of change ($\beta$)	6.192	0.011	542.145	***
	Random intercept variance ($\psi_{\alpha \alpha }$)	13.341	0.624	21.386	***
	Random slope variance ($\psi_{\beta \beta }$)	0.077	0.007	10.554	***
	Covariance ($\psi_{\alpha \beta }$)	0.51	0.052	9.78	***

^***^p < 0.0001

A comparison of the growth parameters across the four groups shows variations in growth patterns in height. Ethiopia captured the initial mean height of 71.38, *p* < 0.0001 with the rate of change 6.11, *p* < 0.0001. India captured the initial mean height of 72.15, *p* < 0.0001 with the rate of change 5.98, *p* < 0.0001. Peru captured the initial mean height of 71.77, *p* < 0.0001 with the rate of change of 6.13, *p* < 0.0001. Vietnam captured the initial mean height of 72.40, *p* < 0.0001 with the rate of change of 6.19, *p* < 0.0001. The variance–covariance structure varies depending on the detected group trajectories. In all groups, the intercept and slope variance were significant. The significant and positive covariance between mean intercept and slope for both Peru and Vietnam groups indicate that children who had a higher initial height tended to be growing at a faster rate. However, the covariance was not significant in the Ethiopian and Indian groups (Ethiopia: $\psi_{\alpha \beta } = 0.061, p = 0.446, India: \psi_{\alpha \beta } = 0.116,{\text{p}} = 0.083$).

### Latent basis growth mixture model with unobserved groups

Individual categorization of trends based on prior information may result in developmental profiles being over-or under-fitted [28]. Instead, in the absence of a priori group classification, the growth mixture model provides an analytical opportunity to identify between different developmental shapes [2]. A growth mixture model can extend a multiple-group approach by introducing latent classes and a probabilistic categorization for each individual [28].

**Fig. 4:**
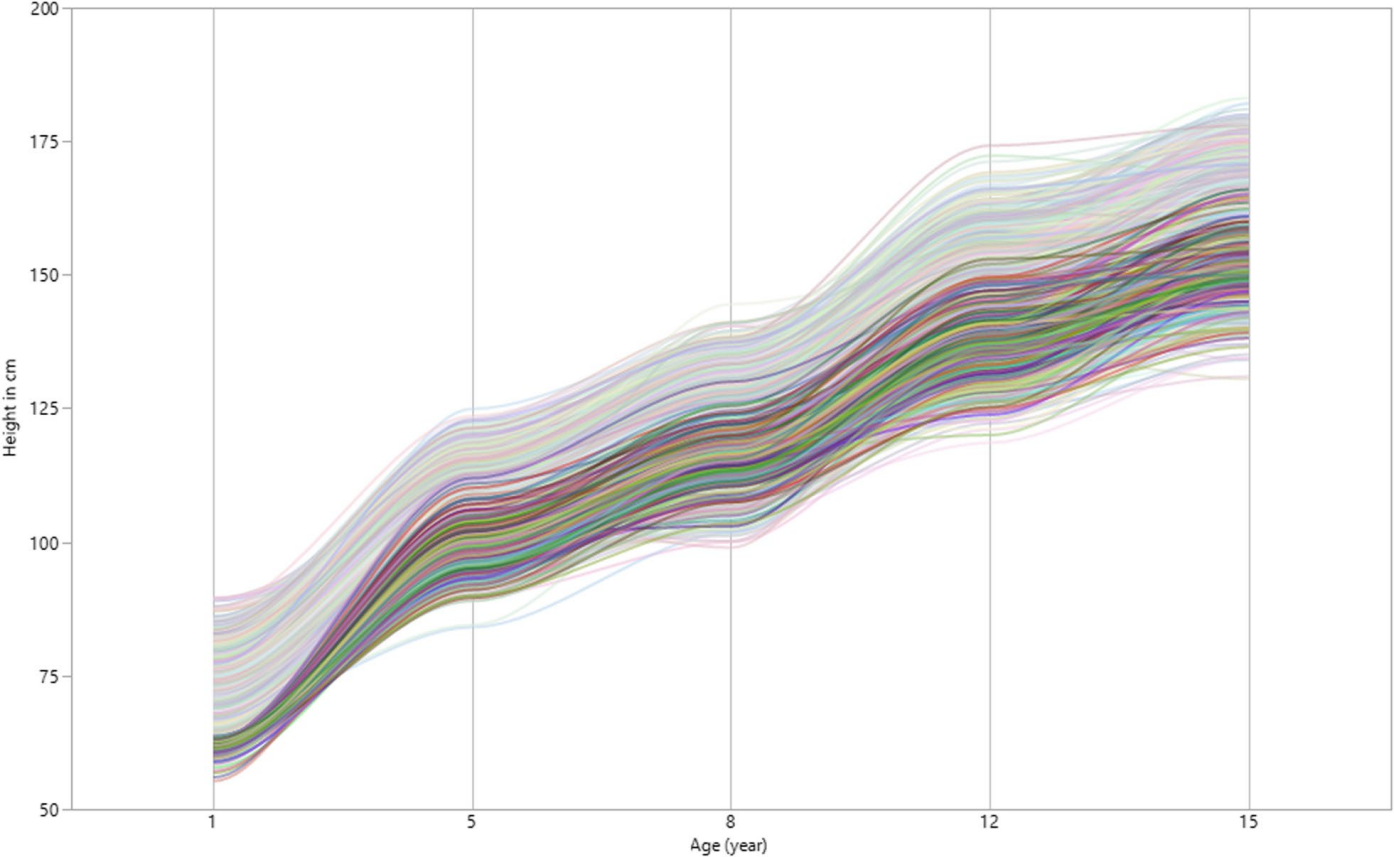
Individuals growth trajectories across five measurement occasions

Figure 4 shows the plot of individual height measurements of the children against time for five waves of data. The visual information displayed in Fig. 4 shows that there is some indication of differences in height growth between and within the children. It also shows nonlinear growth changes in a child’s height over time. The between-individual variability in height growth was small in early childhood and increases with a child's age. Figure 4 also suggests that with a few exceptions, individuals with a lower initial height at age one also exhibited a lower height throughout the measurement occasions and other individuals who started with relatively high initial mean height also grew taller over time. This suggests that there could be latent or unobserved subgroups of individuals who show heterogeneous trajectories. For such instances, a growth mixture model is a reasonable model to investigate individuals’ growth heterogeneity by categorizing them into latent categories with more homogeneous trajectories. We analyzed height data collected from children in four low- and middle-income countries. Height measurements were taken on five occasions. For specification purposes, the first and the last measurement occasions were pre-specified to $\lambda_{1} = 0$ and $\lambda_{5} = 14$ factor loadings to accommodate nonlinearity trajectories.

**Fig. 5:**
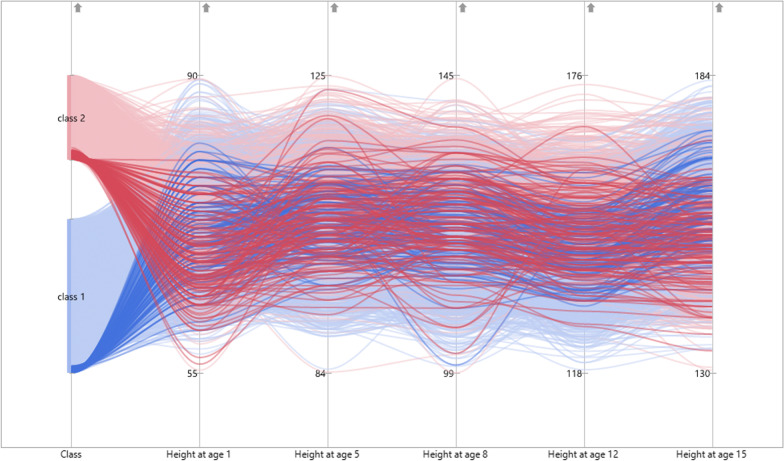
Two-group individual growth trajectories over five time points

The number of latent groups in a growth mixture model is a major research subject. This is accomplished by fitting models with varying numbers of groups in a sequential manner and comparing the fitted models. Three growth mixture models were fitted along with the time-independent covariates to estimate the number of potential latent groups in the height data: a one-group model (H_0_: *g* = 1), a two-group model (H_0_: *g* = 2) and a three-group model (H_0_: *g* = 3). Information criteria–based indices were used to compare these models. Since models with distinct numbers of groups are not nested, a likelihood ratio test is not useable for model comparisons. Instead, Muthen and Muthen (2000) suggest using Bayesian Information Criteria (BIC) to choose the best number of latent groups and a model with a lower value is better-fitting [3, 21]. Table 4 shows the results of model fit indices for three different group growth mixture models. The one-group growth mixture model is the conventional latent growth curve model. The results suggest that the two-group model fits the data much better than the one-group and three-group models as it had a lower BIC value. The two-class model, which is the best-fitting model, divides the data into two groups (Fig. 5). Table 4 shows that the first and the second groups of the growth mixture model comprised 4260 (64.5%) and 2341 (35.5%) of the population, respectively. Females were estimated to comprise 30.3% and 81.6% of groups 1 and 2, respectively, and males were estimated to comprise 69.7%, and 18.4% of groups 1 and 2, respectively.

**Table 4:** The fit of an optimal number of groups for growth mixture models

Group number	Log-likelihood	BIC	Group size (%)
1	− 106,954.4	213,961.7	6601 (100)
2	− 106,920.9	213,929.8	4260 (35.5), 2341 (64.5)
3	− 106,906.1	213,935.3	18.48, 41.65, 39.87

*BIC* Bayesian Information Criteria

The evaluation of group membership predictors and their variations in growth parameters within each group is another important aspect of the growth mixture model. A latent basis growth curve model was employed as a baseline model to capture variations in height over five measurement occasions. The results of parameter estimates of the latent basis growth mixture model with two groups are shown in Table 5. The results indicate that each of the two groups exhibited distinct patterns. It can be seen that a latent basis form fits well both latent groups. Group 1 showed a significant $\left( {\alpha_{\mu } = 72.63,p < 0.0001} \right)$ initial height measurement at age one and a significant rate of change over time $\left( {\beta_{\mu } = 6.27,p < 0.0001} \right)$. Group 2 also showed a significant $\left( {\alpha_{\mu } = 72.75,p < 0.0001} \right)$ initial height measurement at age one and a significant rate of change over time $\left( {\beta_{\mu } = 5.92,p < 0.0001} \right)$. The significant intercept variance (Group 1: $\psi_{\alpha \alpha } = 11.93,p < 0.0001$ and Group 2: $\psi_{\alpha \alpha } = 14.23,p < 0.0001$) and slope variance (Group 1: $\psi_{\beta \beta } = 0.14,p < 0.0001$ and Group 2: $\psi_{\beta \beta } = 0.13,p < 0.0001$) indicate that there were considerable inter-individual variations in the initial level of height measurement and rate of changes across individuals, respectively. For group 1, the parameter values of slope factor loadings were estimated to be $\lambda_{1} = 0\left( {fixed} \right),\lambda_{2} = 5.19,\lambda_{3} = 7.72,\lambda_{4} = 11.03,\lambda_{5} = 14\left( {fixed} \right)$ and for group 2 $\lambda_{1} = 0\left( {fixed} \right),\lambda_{2} = 5.77,\lambda_{3} = 8.54,\lambda_{4} = 12.76,\lambda_{5} = 14(fixed$. The significantly different values of slope factor loadings for groups 1 and 2 indicate that latent group 1 and group 2 have different functional forms of trajectories. For group 1, the non-significant covariance between the mean intercept and rate of growth ($\psi_{\alpha \beta } = - 0.045,p = 0.205$) revealed that there was no association between the two growth components, while it was significant in group 2 ($\psi_{\alpha \beta } = - 0.184,p < 0.0001$), indicating that children with higher initial levels of mean height at age one progressed less rapidly in their growth over time than those with lower initial levels of mean height.

**Table 5:** Parameter estimates of 2‑group latent basis growth mixture model

	Group 1				Group 2			
	Estimate	SE	CR	*p*	Estimate	SE	CR	*p*
Latent variable means								
Intercept mean $\left( {\alpha_{\mu } } \right)$	72.632	0.143	508.509	***	72.745	0.266	273.412	***
Slope $\left( {\beta_{\mu } } \right)$	6.274	0.015	412.753	***	5.919	0.025	234.251	***
Time-invariant covariate								
Intercept < –- Gender (Female)	− 2.12	0.144	− 14.71	***	− 1.661	0.248	− 6.695	***
Slope < –- Gender (Female)	− 0.178	0.015	− 11.481	***	− 0.071	0.024	− 3.023	0.003
Country < –- Ethiopia (Reference group)								
Intercept < –- India	0.01	0.181	0.057	0.954	1.286	0.295	4.359	***
Intercept < –- Peru	− 0.61	0.189	− 3.237	0.001	0.723	0.284	2.547	0.011
Intercept < –- Vietnam	− 0.216	0.184	− 1.171	0.241	1.128	0.278	4.059	***
Slope < –- India	− 0.146	0.019	− 7.537	***	− 0.117	0.028	− 4.168	***
Slope < –- Peru	0.043	0.02	2.109	0.035	0.07	0.027	2.582	0.01
Slope < –- Vietnam	0.092	0.02	4.631	***	0.171	0.026	6.494	***
Latent variance–covariance								
Intercept variance	11.934	0.435	27.427	***	14.232	0.699	20.368	***
Slope variance	0.136	0.005	27.699	***	0.127	0.006	21.32	***
Intercept < –- Slope	− 0.045	0.036	− 1.267	0.205	− 0.184	0.052	− 3.541	***
Slope factor loadings								
Time 1	0 (fixed)				0 (fixed)			
Time 2	5.189	0.009	590.018	***	5.765	0.013	440.033	***
Time 3	7.718	0.009	895.078	***	8.543	0.013	633.347	***
Time 4	11.032	0.009	1217.075	***	12.759	0.012	1032.696	***
Time 5	14 (fixed)				14 (fixed)			

^***^p < 0.0001

The next work is to investigate and evaluate the likely differences in the covariate influences on these two group trajectories. This is accomplished by introducing group-specific time-invariant covariates, creating a conditional growth mixture model, to capture the difference in the group-specific growth factors. This conditional model entails defining analysis of continuous latent growth factors and groups of categorical latent variables on gender and residing country of children. The fitted results of the conditional growth mixture model are given in Table 5. Gender and country differences were found to be associated with the growth factors, but the relationship differed with the trajectory group. For instance, females tended to have lower growth factors compared with their male counterparts in both groups.

Compared with children from Ethiopia, children from Peru and Vietnam tended to exhibit faster growth in height over time: Group 1: ($\beta_{Peru} = 0.04,p = 0.035,\beta_{Viet} = 0.09,p < 0.0001$) and Group 2: ($\beta_{Peru} = 0.07,p = 0.01,\beta_{Viet} = 0.17,p < 0.0001$). In contrast, children from India showed a lower rate of change in both latent groups than that of children from Ethiopia: Group 1: ($\beta_{India} = - 0.15,p < 0.0001$) and Group 2: ($\beta_{India} = - 0.12,p = 0.01,p < 0.0001$). Furthermore, the findings showed that the effects of selected covariates on initial levels of mean height varied throughout latent trajectory groups.

## Discussion

Many analyses of longitudinal data assume that there is a single population on a single trend. Data from a single population assumed that individuals contribute comparable information about a hypothesized growth process. However, individuals may not have similar growth trajectories over time and they can be classified into various groups based on their growth trajectories. Because of the intrinsic heterogeneity found in trajectories of some individual characteristics, the assumption of a single population is not reasonable. In the current study, two mixture modeling approaches were applied to investigate growth heterogeneity in the height of children in four low- and middle-income countries. First, it is when the individual's group membership is known and second when the individual's group membership is unknown, and there are multiple groups with different growth trajectories. The latter technique permits for the evaluation of height change over time while explicitly permitting the latent growth structure to be parameterized with a specific number of groups.

The findings of the study identified that there was a substantial difference in growth parameters across the four observed groups. The differences in growth trajectories were observed among children. Subsequently, we defined an unobserved growth mixture model as an extension of a multiple-group growth model that is useful for analyzing the growth variations in multiple latent or unobserved subgroups. We performed Growth mixture models with one, two and three groups applying a latent basis growth curve model for all groups to estimate the number of potential latent groups. The BIC scores for these group models were evaluated, and the two-group latent basis mixture model was preferred for the current height data. In this model, individuals were categorized into two groups, with Group 1 accounting for 64.5 percent of the total and Group 2 accounting for 35.5 percent. Group 1 had the most male samples (69.7%), and Group 2 had the most female samples (81.6%). Two group trajectories were identified in the previous longitudinal study that employed growth mixture modeling to estimate weight-to-height trajectories in children aged 4 to 12 years [29]. The previous study has investigated sex-specific latent height class trajectories and identified the three-class model for males (low, intermediate and high) and the two-class model for females (low and high) (low and high). The study reported that in both sexes, height was significantly distinct between classes [30].

The results of the study demonstrated that the growth trajectories of children in four low- and middle-income countries were categorized into two different group trajectories. Gender and country differences were found to be associated with the growth factors, but the relationship differed with the trajectory group. Females had lower growth factors in both latent groups compared with males. In both trajectory groups, compared with children from Ethiopia, those from Peru and Vietnam grew taller more quickly over time. Children from India, on the other hand, showed a lower rate of change in both latent groups than children from Ethiopia.

When studying growth trajectories within a population, the growth mixture model provides a lot of potential for identifying group heterogeneity. Growth trajectories were found to be heterogeneous and the study identified two different group trajectories. The mean, variance and weight structures of the two groups were all different.

The limitation of the study is that it focused only on four low and middle-income countries with few covariates. As a result, further study is needed to address these limitations.

## Conclusion

The study identified that the height of children in four low- and middle-income countries showed heterogeneous changes over time with two different groups of growth trajectories. Group 1 had the most male samples, and Group 2 had the most female samples. This study may also provide valuable insights for a better understanding of how to model growth heterogeneity in the context of mixture models.

## Data Availability

The datasets analyzed during the current study are available in the Young Lives study repository, http://www.younglives.org.uk/.
